# Demographic characteristics, site and phylogenetic distribution of dogs with appendicular osteosarcoma: 744 dogs (2000-2015)

**DOI:** 10.1371/journal.pone.0223243

**Published:** 2019-12-30

**Authors:** Joanne L. Tuohy, Marejka H. Shaevitz, Laura D. Garrett, Audrey Ruple, Laura E. Selmic

**Affiliations:** 1 Department of Small Animal Clinical Sciences, Virginia-Maryland College of Veterinary Medicine, Blacksburg, Virginia, United States of America; 2 Department of Veterinary Clinical Medicine, College of Veterinary Medicine, University of Illinois, Urbana, Illinois, United States of America; 3 Department of Comparative Pathobiology, College of Veterinary Medicine, Purdue University, West Lafayette, Indiana, United States of America; 4 Department of Veterinary Clinical Sciences, College of Veterinary Medicine, The Ohio State University, Columbus, Ohio, United States of America; Universite de Nantes, FRANCE

## Abstract

**Objective:**

To report demographic characteristics of a contemporary population of dogs with appendicular osteosarcoma and assess the relationship between demographic characteristics, site distribution, and phylogenetic breed clusters.

**Design:**

Retrospective case series.

**Methods:**

A search of the Veterinary Medical Database was performed for dogs with appendicular osteosarcoma as a new diagnosis. Entries were reviewed for the sex, neuter status, age at diagnosis, breed, affected limb, and tumor location. The reported breed for purebred dogs was used to categorize each dog into one of five phylogenetic groups based on microsatellite analysis.

**Results:**

744 client-owned dogs were included in the study. Study dogs were represented by a male-to-female ratio of 0.95:1.0, the majority of which (80.9%) were neutered. Most dogs were diagnosed between 7–10 years of age. The majority (77.8%) of dogs were large or giant-breed dogs. Purebred dogs comprised 80.4% of the population. The most common purebred breed affected by OS was the Rottweiler (17.1%). The most common phylogenetic group represented was Mastiff-Terrier (M-T, 26.3%). OS was more commonly located in the forelimb (64.2%) versus the hindlimb (35.8%), and the humerus was the most common site (20.9%). The distribution of age groups and tumor locations were significantly different between phylogenetic clusters. The distribution of age groups and neuter status were significantly different between size groups.

**Conclusions and significance:**

The demographic data of canine appendicular OS are similar to previous reports. The data on phylogenetic associations can guide future studies aimed at evaluating the genomic mutations that contribute to OS development and its biological behavior.

## Introduction

Osteosarcoma (OS) is the most common canine primary bone tumor and represents up to 85% of all primary malignant bone tumors in the dog.[1–5] Osteosarcoma of the appendicular skeleton occurs more commonly than OS of the axial skeleton.[3, 6–10] The demographic characteristics from populations of dogs affected by appendicular OS have been extensively described, with some reports dating back decades.[1–5, 11] Studies report appendicular OS to be largely a disease of middle-aged to older dogs with the median age at diagnosis generally ranging from 6 to 9 years.[2–4, 8, 10, 12–16] Males have been over-represented in some reports but this finding has not been supported in other reports.[2, 3, 7–10, 12–18] Endogenous sex hormones may play a role in sarcomagenesis–some studies have identified significantly increased risk of developing OS in neutered compared to intact dogs.[13, 19–21] Appendicular OS has been commonly reported in large and giant breed dogs, with increased incidence of disease in certain breeds including boxers, Great Danes, Rottweilers, Saint Bernards, Irish setters, Doberman pinschers, greyhounds, German shepherds, Irish wolfhounds and Leonbergers.[1, 3, 8, 11–14, 18, 21–26] An increased risk of OS development has been identified with increasing weight, height and age.[13] Appendicular OS occurs predominantly at the metaphyses of long bones and affects the bones of the forelimb more commonly than the hindlimb.[3, 7, 15, 22, 27] The distal portion of the radius and the proximal portion of the humerus are the most commonly affected sites.[2, 3, 7, 14, 16, 18, 20, 25, 28] The pathogenesis of canine OS and underlying factors driving its phenotypic manifestations are not well understood.

Genomic studies have begun to build our understanding of the genomic dysregulation, such as amplification of oncogenes and down-regulation of tumor suppressor genes, that make up the molecular basis of canine OS development.[29–33] The predisposition of certain dog breeds to developing OS suggests a contribution of genetic factors to increased disease risk. A review of genomic analyses in dog breeds described the 5 major dog breed clusters created by microsatellite clustering analysis that assigns groups based on allelic similarities—Ancient-Asian (A-A), Herding-Sighthound (H-S), Modern (Md), Mastiff-Terrier (M-T) and Mountain (Mn).[34] Mapping a disease such as OS across breed clusters can yield valuable information to direct the design of follow-up studies aimed at identifying genomic mutations and subsequent interventional targets, and help identify high risk populations for screening. To the authors’ knowledge, no study has evaluated patterns in demographics and the site distribution of canine OS in relationship to the major phylogenetic dog breed clusters.

The aim of the study was to provide a current description of the demographic characteristics and site distribution of a large contemporary population of dogs with appendicular OS and assess the relationship between demographic characteristics, site distribution, and phylogenetic breed clusters. The information was successfully compiled and presented below.

## Materials and methods

### Case selection

A Veterinary Medical Database (VMDB, http://www.vmdb.org. The VMDB does not make any implicit or implied opinion on the subject of the study.) search was performed to identify medical records of dogs seen at participating veterinary teaching hospitals with a diagnosis of OS from January 1^st^ 2000 to December 31^st^ 2015. Appendicular OS was defined to include dogs with OS of the scapula, humerus, shoulder, radius, ulna, femur, tibia, metacarpals, metatarsals, sesamoids, patella, stifle, carpal bones, tarsal bones or phalanges. Records were excluded if they represented a diagnosis of a non-appendicular OS, or if the location of the lesion was not identified in the record. The earliest visit for each individual patient was used for analyses.

### Database record review

Demographic information obtained from the database entries included age category at diagnosis, sex, neuter status, and breed. Age categories in the VMDB were defined as 6–12 months; 1–2 years; 2–4 years; 4–7 years; 7–10 years; 10–15 years; and >15 years. Dogs were assigned to individual age categories by the institutions contributing to the VMDB. Dogs at 6–12 months were categorized as “Junior”, at 1–3 and 2–4 years as “Adult”, at 4–7 years as “Mature”, at 7–10 and 10–15 years as “Senior”, and at >15 years as “Geriatric” based on published canine life stage guidelines.[35] Data on neuter status were categorized into the categories of “Female Intact”, “Female Spayed”, “Male Intact”, “Male Castrated”. Data indicating the limb affected by the lesion (forelimb vs. hindlimb) and the affected bone or bones were collected. For purebred dogs with assigned breeds, one author (MHS) assigned each dog to a phylogenetic cluster based on microsatellite analysis performed using 130 dog breeds.[36] One author (MHS) assigned a size category (small-medium, large, giant) to each dog according to American Kennel Club (AKC) breed size standards if weight definitions for that particular breed were available.[37] When the AKC breed standards did not include weight definitions for a particular breed, a single online resource (Hill’s) was used to obtain average breed weights, which were then used to assign size categories to those breeds.[38] The assignment of size category according to breed was performed to reduce biases such as the bias of obesity or of being underweight, which cannot be assessed accurately using the VMDB, thus avoiding the assignment of dogs of the same breed into different size categories. Of the 56 breeds represented, 23 were categorized into breed size using AKC weight standards, one breed was categorized using a combination of AKC breed standards and the Hill’s online resource, and the remaining breeds were categorized solely using the Hill’s online resource.

### Statistical analysis

Statistics were calculated using SAS software, Version 9.4 of the SAS System for PC. (Copyright 2013 SAS Institute Inc. SAS and all other SAS Institute Inc. product or service names are registered trademarks or trademarks of SAS Institute Inc., Cary, NC, USA.) Descriptive statistics were calculated including mean, standard deviation and range to describe the age and weight (PROC UNIVARIATE). The categorical variables of breed and anatomic site was displayed in a tabular form and described with frequencies and percentages (PROC FREQ). Chi square tests were used to assess for associations between age, sex, neuter status, affected limb (forelimb vs. hindlimb), tumor locations and grouping of phylogenetic clusters or size category.

## Results

**Fig 1:**
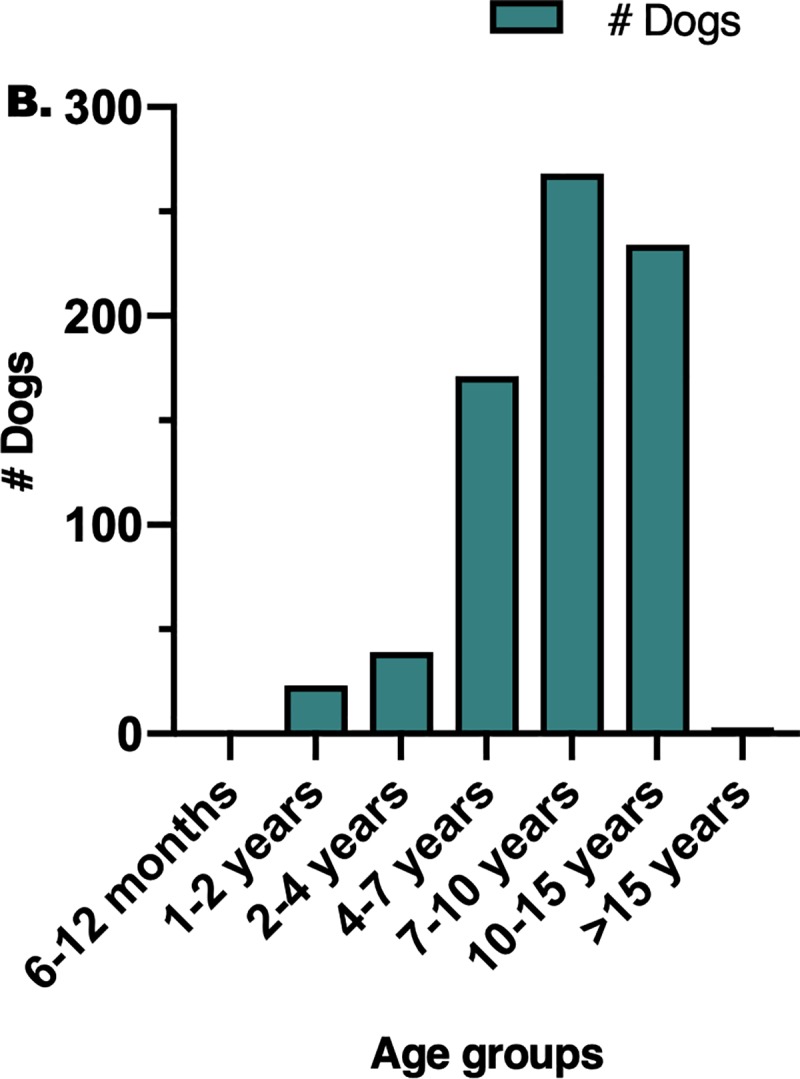
Bar graph to show the distribution of age groups in the entire study population.

There were 11,848 records of visits identified for dogs affected with OS in the VMDB database, which included records from 15 veterinary teaching hospitals. Of those records, 8338 were visits for appendicular OS in 744 dogs that were subsequently included in this study. The other 3614 visits were for dogs that met exclusion criteria of osteosarcoma at non-appendicular sites (2951) or where the affected site was not recorded (559). There were 381 female dogs (51.2%) and 363 male dogs (48.8%) in the study population, representing a male-to-female ratio of 0.95:1.0, and the majority of dogs were neutered (602/744; 80.9%). The most common age category at diagnosis was 7–10 years (36.0%) followed by 10–15 years (31.5%), both categories fell under the “Senior” classification, representing middle-aged to older dogs (Fig 1). The majority of dogs were large-breed (49.5%) or giant-breed (28.3%). These data are displayed in Table 1.

**Table 1:** Characteristics (sex, neuter status, age and weight) of 744 dogs with appendicular OS included in the study.

	# dogs	% study population
**Sex**		
Female	381	51.2
Male	363	48.8
**Neuter status**		
Female spayed	334	44.9
Female intact	47	6.3
Male castrated	268	36.0
Male intact	95	12.8
**Age categories**		
6–12 months	1	0.1
1–2 years	23	3.1
2–4 years	39	5.2
4–7 years	171	23.0
7–10 years	268	36.0
10–15 years	234	31.5
>15 years	3	0.4
Unknown	5	0.7
**Size categories**		
Small/medium	19	2.6
Large	368	49.5
Giant	211	28.3
Unknown	146	19.6

Purebred dogs made up 80.4% of the study population. There were 56 different breeds represented by 598 dogs, 3 purebred dogs were coded as other purebred and likely represented rare purebreeds (0.4%). There were 143 mixed-breed dogs (19.2%). The most common purebred dogs affected by OS were the Rottweiler (17.1%), golden retriever (11.8%), Labrador retriever (10.9%), Doberman pinscher (5.7%), greyhound (5.1%), German Shepherd (4.7%), Saint Bernard (3.0%), Irish wolfhound (2.2%), Great Dane (1.9%), Great Pyrenees (1.3%), and Irish setter (1.3%). Forty-four different breeds made up the remaining 15.8% of the population of dogs (Table 2). Five hundred and forty-six of the 598 purebred dogs represented 36 distinct breeds that could be categorized into these 5 phylogenetic groups–Ancient-Asian (A-A), Herding-Sighthound (H-S), Mastiff-Terrier (M-T), Modern (Md), and Mountain (Mn). Fifty-two purebred dogs represented 20 breeds for which no phylogenetic group assignment was available. The most common phylogenetic group represented was M-T (26.3%), followed by Mn (22.3%), and Md (12.5%) (Table 3).

**Table 2:** Breeds of dogs with appendicular OS included in the study.

Breeds represented in study population	# dogs	% of study population
Rottweiler	127	17.1
Golden retriever	88	11.8
Labrador retriever	81	10.9
Doberman pinscher	42	5.7
Greyhound	38	5.1
German shepherd	35	4.7
Saint Bernard	22	3.0
Irish wolfhound	16	2.2
Great Dane	14	1.9
Great Pyrenees	10	1.3
Irish setter	10	1.3
Siberian husky	8	1.1
Newfoundland	7	0.9
Boxer	7	0.9
Dalmatian	6	0.8
Rhodesian ridgeback	6	0.8
Alaskan malamute	5	0.7
Australian shepherd	5	0.7
Mastiff	5	0.7
Old English sheepdog	5	0.7
American cocker spaniel	4	0.5
Bullmastiff	4	0.5
Poodle, standard	4	0.5
Shetland sheepdog	4	0.5
Bernese mountain dog	3	0.4
Collie	3	0.4
English setter	3	0.4
Other breeds (2 or fewer represented)	36	4.9
Mixed breed dog	143	19.2
Other pure breed	3	0.4

**Table 3:** Distribution of breeds within phylogenetic clusters, number of dogs, and overall % study population represented per cluster.

Phylogenetic group	Number of dogs and % of study population in each phylogenetic group	Breeds represented in each phylogenetic group	Number of dogs represented by individual breeds
M-T	196; 26.3%	Boxer	7
		Bulldog	1
		Bullmastiff	4
		Golden retriever	88
		Jack Russell terrier	2
		Labrador retriever	81
		Mastiff	5
		Newfoundland	7
Mn	166; 22.3%	Bernese mountain dog	3
		Great Dane	14
		Rottweiler	127
		Saint Bernard	22
Md	93; 12.5%	American cocker spaniel	4
		Doberman pinscher	42
		English springer spaniel	2
		German shepherd dog	35
		German shorthaired pointer	2
		German wirehaired pointer	1
		Poodle, standard	4
		Portuguese water dog	1
		Schnauzer, giant	1
H-S	75; 10.1%	Australian shepherd	5
		Border collie	1
		Borzoi	2
		Collie	3
		Greyhound	38
		Irish wolfhound	16
		Kuvasz	1
		Old English sheepdog	5
		Shetland sheepdog	4
A-A	16; 2.2%	Afghan hound	1
		Alaskan malamute	5
		Samoyed	1
		Shar-Pei	1
		Siberian husky	8
NP	55; 7.4%	Airedale terrier	2
		American Staffordshire terrier	1
		Anatolian shepherd	1
		Australian heeler	1
		Beauceron	1
		Black and tan coonhound	1
		Bouvier des Flandres	1
		Chesapeake Bay retriever	2
		Curly-Coated retriever	1
		Dalmatian	6
		English foxhound	1
		English setter	3
		Gordon setter	1
		Great Pyrenees	10
		Irish setter	10
		Lhasa apso	1
		Leonberger	1
		Norwegian elkhound	1
		Other purebred canine	3
		Rhodesian ridgeback	6
		Skye terrier	1

A-A = Ancient-Asian; H-T = Herding-Sighthound; M-T = Mastiff-Terrier; Md = Modern; Mn = Mountain. NP = no assignable phylogenetic cluster.

Appendicular OS was located in the forelimb in 478 cases (478/744; 64.2%) and the hindlimb in 266 cases (266/744; 35.8%). Specific locations for OS in this study included humerus, radius, ulna, radius/ulna, femur, tibia, carpus, tarsus, scapula, metacarpus, metatarsus, shoulder, and stifle. The “radius/ulna” location included cases where the records were unclear whether the radius or ulna was affected. Cases identified as shoulder (15/744) and stifle (15/744) were excluded from the analysis of tumor location, as a specific bone was not identified. The most frequent single bone affected was the humerus (149/714; 20.9%), followed by the femur (132/714; 18.5%), and radius (101/714; 14.2%) (Table 4).

**Table 4:** Location of OS in study population.

Tumor locations	Number	% of affected locations
Humerus	149	20.9
Femur	132	18.5
Radius	101	14.1
Tibia	96	13.5
Ulna	65	9.1
Scapula	61	8.5
Radius/ulna	54	7.6
Carpus	25	3.5
Tarsus	18	2.5
Metacarpus	8	1.1
Metatarsus	5	0.7

**Fig 2:**
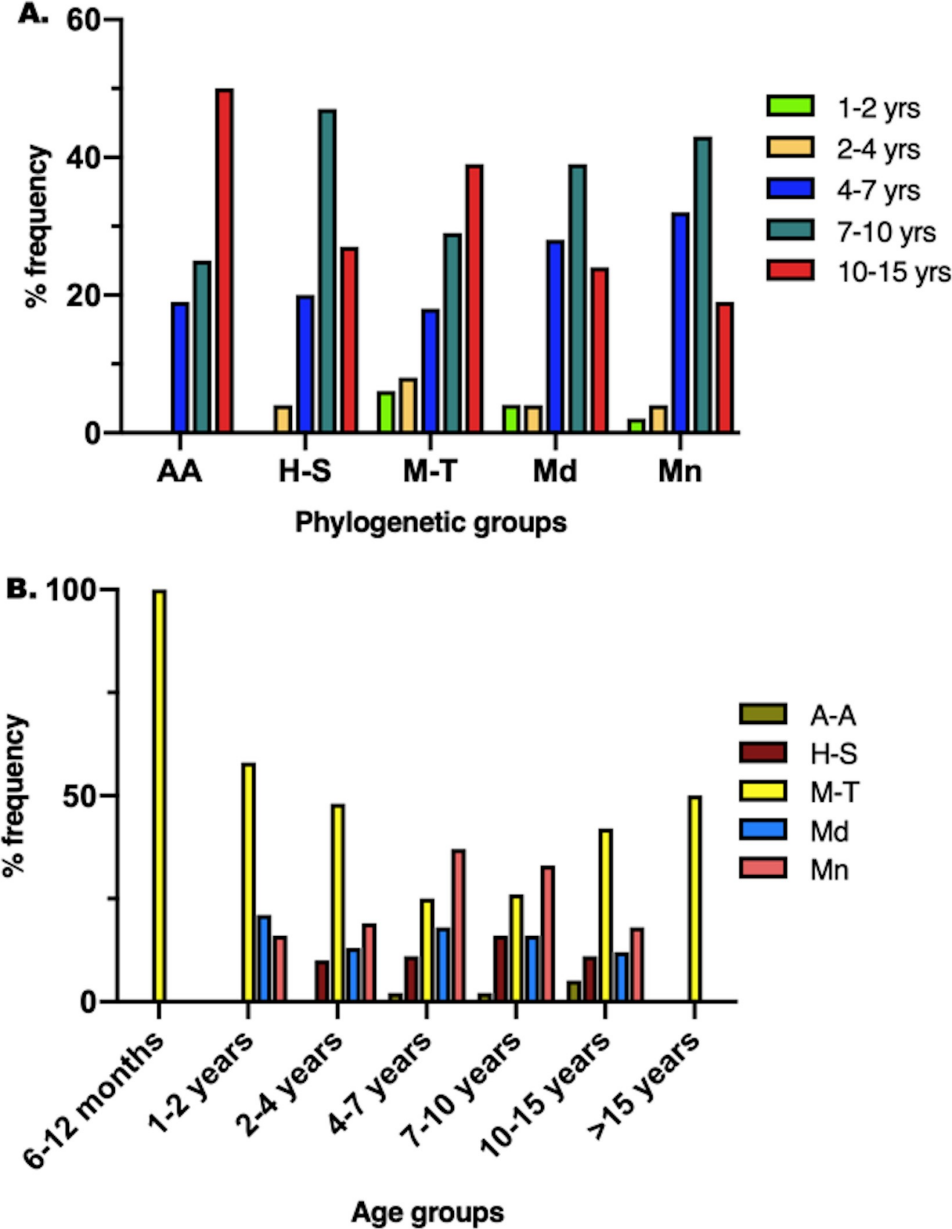
**Bar graphs showing the distribution of: (A) age groups between phylogenetic groups, (B) phylogenetic groups between different age groups.** (A) The distribution of age categories was significantly different between the phylogenetic clusters (p = 0.0006). Either the 7–10 years or the 10–15 years category was the most frequently represented in all phylogenetic clusters. The 6–12 months and >15 years age categories each had 1 dog in the M-T cluster, and are not graphically represented. (B) The distribution of phylogenetic clusters was significantly different between age categories (p = 0.0006). The Mn cluster was the most frequently represented cluster among the 4–7 and the 7–10 year old dogs. The M-T cluster was the most frequently represented cluster among all other clusters. A-A = Ancient-Asian; H-T = Herding-Sighthound; M-T = Mastiff-Terrier; Md = Modern; Mn = Mountain.

The distribution of age categories was significantly different between the phylogenetic clusters, and the distribution of phylogenetic clusters was significantly different between age categories (p = 0.0006) (Fig 2). Senior dogs that were between 10–15 years old were the most frequently represented age category in the A-A and M-T clusters (50.0%, 38.8% respectively), and senior dogs that were 7–10 years old were the most frequently represented age category in the H-S, Md and Mn clusters (46.7%, 38.7%, 43.4% respectively). The phylogenetic cluster most commonly represented in the 7–10 years and 10–15 years age categories were Mn (32.3%) and M-T (42.5%) respectively. The phylogenetic cluster most commonly represented in the 1–2 years age category was M-T (57.9%).

**Fig 3:**
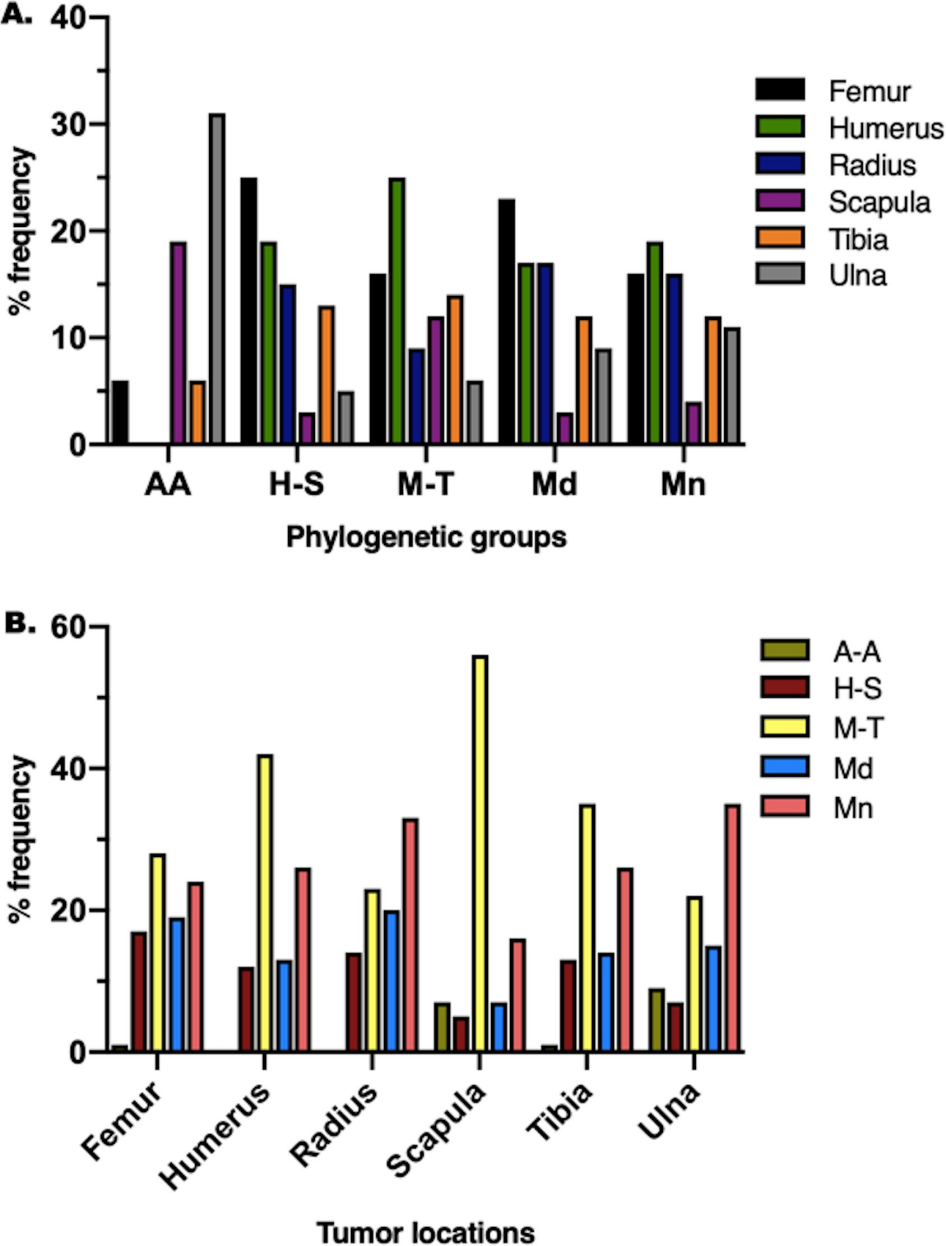
**Bar graphs showing the distribution of: (A) tumor locations between phylogenetic groups, (B) phylogenetic groups between different tumor locations.** (A) The distribution of tumor location was significantly different between the phylogenetic clusters (p = 0.008). The humerus was the most common tumor location overall, and was the most frequently represented in the M-T and Mn clusters. (B) The distribution of phylogenetic clusters was significantly different between tumor locations (p = 0.008). The M-T cluster was the most frequently represented in tumors of the femur, humerus, scapula, and tibia.

The distribution of tumor location was significantly different between phylogenetic clusters, and the distribution of phylogenetic clusters was significantly different between tumor locations (p = 0.008) (Fig 3). The humerus was the most common location in the M-T (25.0%) and Mn (18.7%) clusters. The femur was the most common location in the H-S (25.3%) and Md (22.6%) clusters, with the ulna being most common in the A-A (31.3%) cluster. The M-T cluster represented the most common cluster that developed humeral lesions (41.2%) and femoral lesions (27.9%), whereas the Mn cluster represented the most common cluster that developed radial lesions (32.5%). No significant distribution associations were found between phylogenetic cluster and sex (male/female; p = 0.55), neuter status (p = 0.61), or affected limb (fore / hind; p = 0.38).

**Fig 4:**
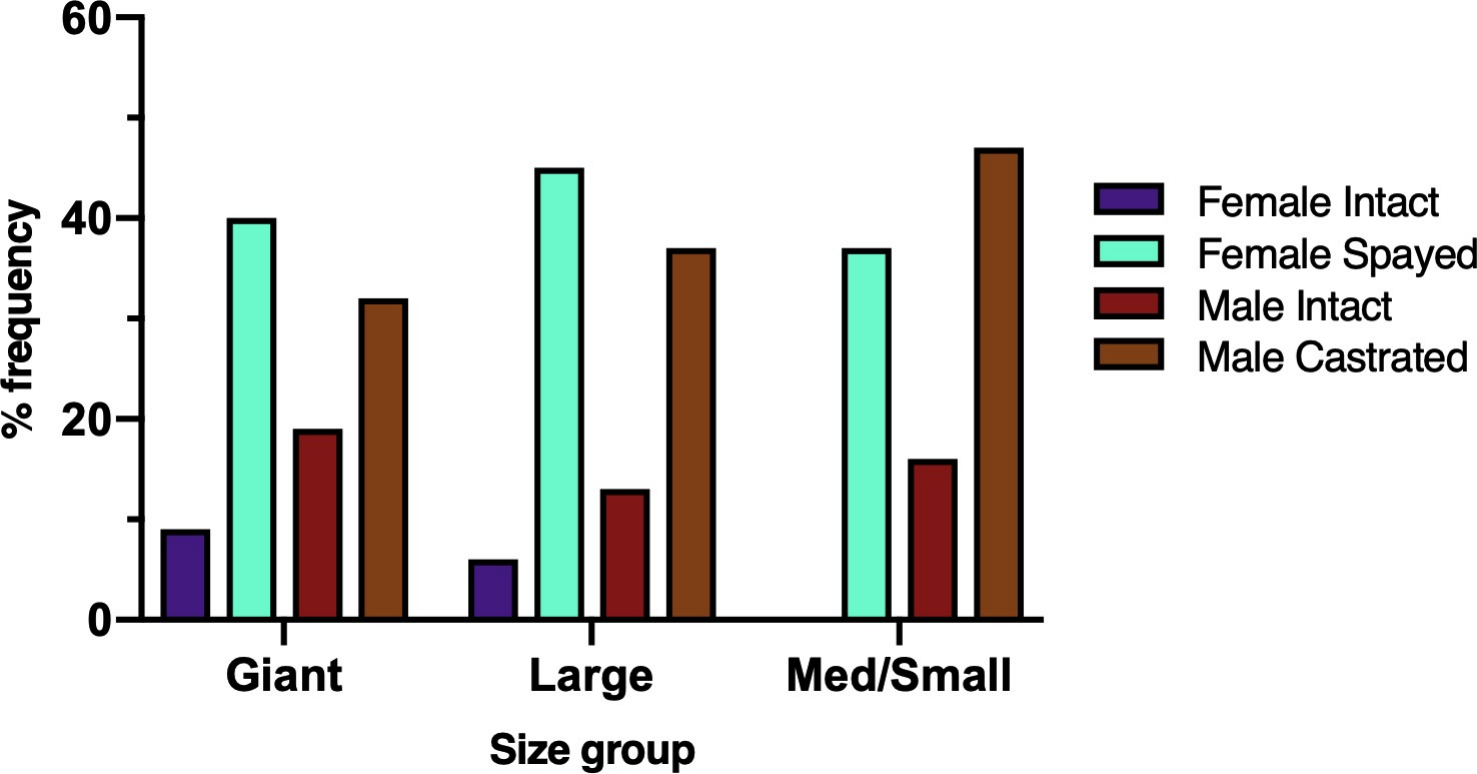
Bar graphs showing the distribution of neuter status between size groups. The distribution of neutered / intact dogs was significantly different between the size categories of dogs (p = 0.003). Female spayed dogs were the most frequently represented among giant and large breed dogs, whereas male castrated dogs were most frequently represented among medium/small dogs.

The distribution of neutered / intact dogs was significantly different between the size categories of dogs (p = 0.003) (Fig 4). Female spayed dogs were most commonly represented among large (44.6%) and giant (40.3%) breed dogs, and male castrated dogs were most commonly represented among medium / small breed dogs (47.4%).

**Fig 5:**
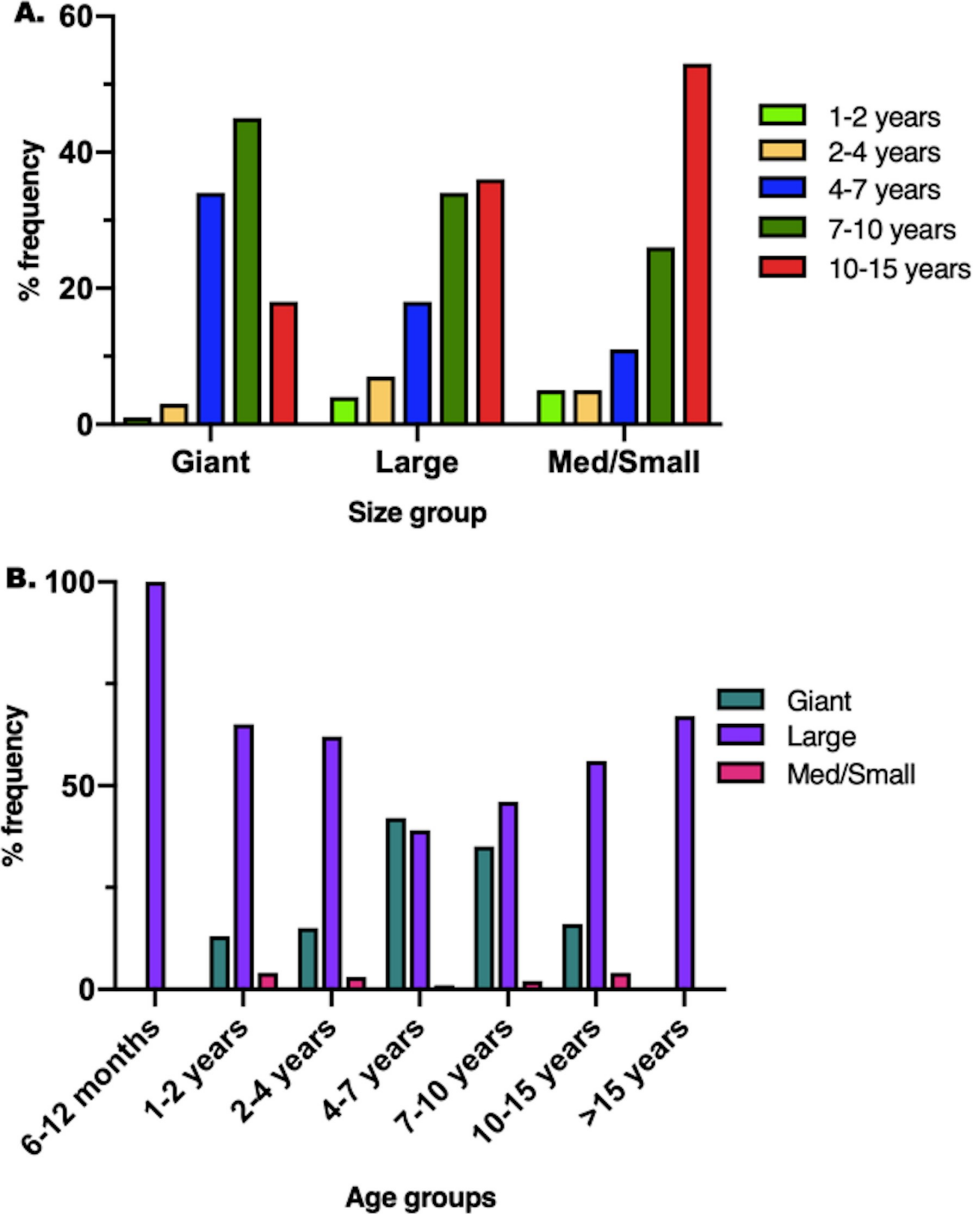
**Bar graphs showing the distribution of: (A) age groups between different sizes, (B) sizes between different age groups.** (A) The distribution of age was significantly different between the size groups (p = 0.0001). The 10–15 year old dogs were the most frequently represented group in large and medium/small dogs, whereas the 7–10 year old dogs were the most frequently represented group in giant breed dogs. The 6–12 months and >15 years age categories each had 1 dog in the large size category and are not graphically represented. (B) The distribution of size was significantly different between age groups (p = 0.0001). Large breed dogs were the most commonly represented group in all age groups except for the 4–7 year old dogs, where giant breed dogs were most common.

The distribution of age was significantly different between the various sizes of dogs, and the distribution of size was significantly different among the various age groups (p = 0.0001) (Fig 5). In giant breed dogs, the most common affected age group was 7–10 years (44.6%), in large breed dogs, the most common affected age group was 10–15 years (35.6%). Large breed dogs were the most commonly represented size in the 7–10 year old dogs (46.3%), the 10–15 year old dogs (56.0%), and the 1–2 year old dogs (65.2%), whereas giant breed dogs were the most commonly represented size in the 4–7 year old dogs (41.5%). No significant distribution associations were found between size and sex (male / female; p = 0.13), affected limb (fore / hind; p = 0.51), or tumor location (p = 0.29).

## Discussion

This study included 744 dogs with appendicular OS, and the demographic data of these dogs are similar to previous studies.[2–4, 7, 12–14, 17, 21–23, 25, 26] This study found the distributions of age and of tumor location to be significantly different between phylogenetic clusters, and the distributions of phylogenetic clusters to be significantly different between age groups and between tumor locations. There was also significant variation in neuter status and age distributions between the different sized dogs, and there was significant variation in distributions of dog sizes between the different ages and neuter categories of dogs.

Consistent with previous reports, this study found that appendicular OS was most commonly diagnosed in middle-aged to older, large and giant breed dogs, and affected the forelimb more frequently than the hindlimb.[3, 7, 15] The 3 most common purebred dogs that were diagnosed with OS in this study were the Rottweiler (Mn cluster), golden retriever and Labrador retriever (both in M-T cluster). The most common phylogenetic group represented was M-T, followed by Mn. There potentially could exist mutational similarities contributing to the development of OS between M-T and Mn clusters which may be revealed with future genomic mapping studies.

The humerus was the most common site for canine appendicular OS in this study, consistent with some reports.[7, 20] However, the radius has been more frequently reported as the most common site for canine OS.[2, 3, 14, 16, 18, 25, 26, 28] The category of “radius/ulna” as a tumor location in this study may have affected the accuracy of the evaluation of tumor location frequency, as this category included tumors whose bone of origin (radius versus ulna) could not be clearly distinguished from the medical record, and thus were not included in the analysis for location frequency. Exclusion of the 54 cases arising in this area may have led to under-estimation of the distal radius as an anatomic site.

The ratio of male-to-female dogs affected by appendicular OS in this study was 0.95:1.0. This differs from previous findings in some papers of an increased prevalence in male dogs for the disease, with studies reporting a male-to-female ratio ranging from 1.2–1.5:1.0.[2, 3, 14] A more recent study reported a hazard ratio of 0.7 for bone tumors in female compared to male dogs.[12] However, a higher male prevalence for OS is not a consistent observation, with some studies, similar to this study, reporting no significant difference in sex predilection for canine OS.[13, 19, 20] Previous studies have identified an association between neutering and development of sarcoma.[13, 19–21] The current study did not evaluate for correlation between neuter status and development of OS, thus no conclusions can be made as to whether the prevalence of neutered study dogs (81%) correlates with risk of developing OS.

Purebred large breed dogs formed the majority of this study population with the Rottweiler, golden retriever, Labrador retriever, Doberman pinscher, and greyhound being the most common breeds represented. Increased frequency of OS in these breeds has been previously reported.[3, 8, 12, 18, 22, 24] A recent genomic study identified 33 non-overlapping loci associated with OS in three commonly affected dog breeds (Irish wolfhound, greyhound, Rottweiler), and these loci are associated with 50–80% OS risk in these breeds.[33] A single autosomal genetic risk factor for OS has been identified in Scottish deerhounds.[32, 39] These studies, together with a study documenting that breed-based genetic backgrounds affect the tumor karyotypes in canine OS, suggest a strong contribution of the genetic background to the development of the OS phenotype in these breeds.[30] However, there is also argument that the prevalence of OS in large and giant breed dogs may be due to size instead of breed—a case in point being that even though greyhounds and whippets are in the same phylogenetic microsatellite cluster, disease prevalence is very different between the 2 breeds, with the greyhound much more commonly affected.[40] A multifactorial contribution to OS development is likely, instead of a single genetic cause.

The M-T phylogenetic cluster was the most commonly represented cluster among 10–15 year old dogs, and these 10–15 year old dogs were in turn the most frequently represented age group within the M-T cluster. The M-T cluster includes breeds such as the boxer, bulldog, bull terrier, mastiff, and small breed dogs such as the Boston terrier, Australian terrier, Yorkshire terrier making up approximately 47% of the breeds within the cluster. Interestingly, the M-T cluster also was the most commonly represented cluster among 1–2 year old dogs. A bimodal age distribution has been reported for canine OS, with a small early peak in incidence between 18–24 months.[11] Future studies to illuminate the biology of OS in younger dogs can include a focus on breeds within the M-T cluster, and such studies potentially increase the comparative relevance of the dog as a model for human OS, which has a peak incidence in adolescent adults.[41] The Mn cluster was the most commonly represented one among 7–10 year old dogs and had the highest percentage of this age group within the cluster. The Mn cluster includes 4 breeds—Bernese mountain dog, Saint Bernard, Great Dane, and Rottweiler. The humerus was the most common tumor location in the M-T and Mn clusters. The M-T cluster was most frequently represented among humeral as well as femoral lesions, whereas the Mn cluster was most frequently represented among radial lesions. Humeral canine OS lesions have been associated with a poorer prognosis, and the factors that dictate the biological behavior of humeral OS are currently undetermined.[42–44] Future genomic studies to define the factors contributing to the poorer prognosis associated with humeral OS lesions can potentially benefit from targeting the M-T and Mn clusters.

Seven to ten year old dogs were the most commonly represented age group in giant breed dogs affected by OS, whereas 10–15 year old dogs were the most frequently represented group in large breed dogs. Giant breed dogs were the most commonly represented group in 4–7 year old dogs. These findings are in agreement with previous reports observing that giant breed dogs exhibit younger age of OS onset.[11, 45] A potential reason for this observation is that giant breed dogs have lower average life expectancies compared to smaller breed dogs, hence onset of geriatric diseases could occur at a correspondingly younger age.[46] To the authors’ knowledge, there is no evidence to suggest a difference in the genomic signature of OS in giant breed dogs that would directly result in an earlier onset of disease.

There are some potential limitations to this study that should be considered when interpreting the results. A potential selection bias could have been created from utilizing data from the VMDB, which includes cases diagnosed in academic veterinary teaching hospitals and does not include cases from private practice referral and primary care clinics. Another potential limitation is the exclusion of mixed breed dogs from the phylogenetic analyses. However, the possibility of mixed breed dogs sharing the same genetic mutations as purebred dogs has been postulated.[34] Future studies could utilize the wealth of information afforded by mixed breed dogs by comparing their allelic patterns with purebred dogs to narrow down regions of shared genetic mutations causing OS development. The VMDB data have some inherent limitations that need to be considered, one of which was the inability to extract specific information regarding the type of diagnostic tool used to confirm OS. With limb amputation and adjuvant chemotherapy as standard of care therapy for OS, a large proportion of the cases would by default have histologic confirmation of OS. However, there may remain a small proportion of cases which were diagnosed based on imaging findings of an aggressive bone lesion.

## Conclusion

Appendicular OS was diagnosed most frequently in middle-aged to older, large and giant breed dogs, and affected the forelimb more frequently than the hindlimb. The humerus was the most commonly affected site, and the Rottweiler was the most commonly affected purebred dog. There were significant differences in distribution patterns between age, tumor location, and phylogenetic clusters. There were also significant variations in distribution patterns between neuter status, age, and dog sizes. These data can guide future studies aimed at evaluating the genomic mutations that contribute to the pathogenesis of OS, its phenotypic manifestations, and its biological behavior.
